# Experimental reproduction of White Feces Syndrome in whiteleg shrimp, *Penaeus vannamei*

**DOI:** 10.1371/journal.pone.0261289

**Published:** 2021-12-23

**Authors:** Luis Fernando Aranguren Caro, Hung N. Mai, Roberto Cruz-Florez, Frances Laureen Agcalao Marcos, Rod Russel R. Alenton, Arun K. Dhar

**Affiliations:** Aquaculture Pathology Laboratory, School of Animal and Comparative Biomedical Sciences, The University of Arizona, Tucson, Arizona, United States of America; National Cheng Kung University, TAIWAN

## Abstract

White Feces Syndrome (WFS) is an emergent disease of penaeid shrimp (*Penaeus monodon* and *P*. *vannamei*) that is identified by the presence of floating white fecal strings on pond water in grow-out ponds. Although the clinical manifestations of WFS are well defined, the underling etiology remains obscure. WFS has been associated with several enteric pathogens, including *Enterocytozoon hepatopenaei* (EHP). The association is based on studies that found areas where WFS has been reported, the prevalence and severity of EHP infection are high. In this study, we describe an experimental reproduction of WFS in *P*. *vannamei* pre-infected with EHP and challenged with a unique isolate of *Vibrio parahaemolyticus* isolated from the gastrointestinal tract of a shrimp displaying WFS. Upon laboratory challenge, shrimp displaying white fecal strings and white discoloration of the gastrointestinal tract were analyzed by histopathology, *in-situ* hybridization and quantitative PCR. Histological analysis confirmed the lesions of EHP and septic hepatopancreatic necrosis in the hepatopancreas of shrimp exposed to both pathogens. Quantitative PCR showed shrimp infected with both EHP and *V*. *parahaemolyticus* had a significantly higher load of EHP compared to shrimp infected with EHP alone. This is the first demonstration of experimental reproduction of WFS under laboratory conditions when animals are infected with EHP and *V*. *parahaemolyticus* concurrently. The data revealed a synergistic relation between EHP and *V*. *parahaemolyticus* isolate that led to the manifestation of WFS. We propose the gross signs of WFS can be used as an indicator of the presence of EHP infection in association with a particular strain of an enteric *Vibrio* spp. in countries where EHP is endemic.

## Introduction

The shrimp gut microbiota is fundamental to the host’s nutrition, growth, pathogen resistance, and maintenance of the internal homeostasis [1, 2]. Feeding characteristics of shrimp, such as actively grazing and cannibalism, make the host vulnerable to pathogen invasion. Colonization by alternative microorganism, destabilize the intestinal microbiota which leads to infections and co-infections in a taxonomically diverse gastrointestinal (GI) tracts [1, 2]. Although there is limited information on specific immune mechanisms provided by the shrimp gut microbiota there are evidences of its interplay with the digestive activities that directly affect shrimp growth and severity of diseases. Likewise, new emerging diseases were found to target the shrimp hepatopancreas causing growth inhibition, size disparity, loss of appetite and chronic mortality [3]. Recently identified shrimp diseases that exhibit these clinical signs are the hepatopancreatic microsporidiosis (HPM) caused by *Enterocytozoon hepatopenaei* (EHP), and White Feces Syndrome (WFS) whose causative agent or agents remain unidentified.

HPM is a disease of the hepatopancreas in penaeid shrimp caused by several species of microsporidians including EHP. This microsporidian species has been found in several Asian countries such as China, Vietnam, Malaysia, Indonesia, Thailand, and India [4–6]. Recently, EHP has been reported in the Western Hemisphere in South America [7, 8]. The main clinical sign of EHP at a farm level is growth retardation [9], leading to an increased size variability and feed conversion rate (FCR). In advanced stages of the disease, EHP-infected shrimp typically display soft shells and chronic mortalities [10]. EHP has been reported in several species of farmed penaeid shrimp including black tiger prawn *P*. *monodon* [4], *P*. *vannamei* [11], and *P*. *stylirostris* [12, 13]. EHP is an intracellular parasite that proliferates within the cytoplasm of the affected tubule epithelial cells in the hepatopancreas including F cells, B cells, and R cells [13]. The histology of infected tissues reveals the presence of basophilic inclusion bodies within the cytoplasm with rounded or irregular shapes in the hepatopancreas epithelial cells that corresponds to the plasmodium stage. Additional histological lesions include mild to severe sloughing of the tubular epithelial cells, low lipid droplets, and the presence of released spores into the lumen of the hepatopancreas tubules [9, 10].

At a farm level, WFS manifests as white fecal strings floating along the surface water in grow-out ponds. Shrimp in these ponds display unusual clinical signs such as poor growth rate, size disparity, gastrointestinal tract with yellowish to whitish discoloration, softshell, and chronic mortalities [6, 8, 14]. Although association between EHP and WFS have long been suggested, no studies have successfully reproduced this syndrome in a controlled laboratory setting. Even when severe EHP infections occur WFS does not always manifest [15], these observations suggests that WFS is the result of an interplay of more than one microorganism and the other causative agent(s) are yet to be identified. A strong association between WFS and EHP was reported in EHP-endemic regions in SE Asia [6] and Venezuela [8]. In these cases, WFS and EHP were detected along another opportunistic pathogen, *Vibrio* spp., that cause septic hepatopancreatic necrosis (SHPN) [5, 6, 10].

In this study, we isolated and identified a strain of *Vibrio parahaemolyticus* as the missing link between EHP and WFS. Co-infecting EHP-infected shrimp by an immersion challenge with a unique strain of *V*. *parahaemolyticus* that was isolated from gut of a shrimp (*P*. *vannamei*) displaying WFS allowed us to provide the first experimental evidence of WFS reproduction. To our knowledge, this is the first demonstration of the reproduction of WFS under controlled laboratory conditions using two unique pathogens.

## Materials and methods

### Shrimp

Specific pathogen free (SPF) *P*. *vannamei* were obtained from the Oceanic Institute (Oahu, Hawaii). For at least two consecutive years, this population has tested negative by PCR for all OIE-listed pathogens as well as OIE non-listed pathogens including EHP. The bioassay was carried out in the Aquaculture Pathology Laboratory of the University of Arizona. All procedures for the sampling and euthanasia of shrimp were performed following the guidelines established by the American Veterinary Medical Association (AVMA) [16].

### Isolation of *Vibrio parahaemolyticus* from *P*. *vannamei* shrimp displaying WFS and inoculum preparation

The gastrointestinal (GI) tract of a juvenile *P*. *vannamei* shrimp displaying WFS in a grow out pond was dissected, and a small section of the GI tract was directly plated on a Thiosulfate Citrate Bile Salts Sucrose (TCBS) (Difco^TM^) plate and incubated at 30 ^0^C for 24 hr. After an overnight growth, only sucrose negative Colony Forming Units (CFU) were present in the plate. A CFU was picked and plated on a Tryptic Soy Agar (Sigma Aldrich) plate containing 2.5% NaCl (TSA+) for further maintenance of the culture. The bacterial identification was carried out by PCR amplification and sequencing of the 16S rRNA region [17]. In addition, PCR detection of the *ToxR* gene and PirAB^*vp*^ genes were performed following previously published protocols [18, 19]. To prepare the inoculum for the experimental infection, Tryptic Soy Broth (TSB) (Difco^TM^) supplemented with 2% NaCl (TSB+) was used for culturing the isolate at 30°C overnight with gentle shaking (100 rpm) to reach 1.5 × 10^9^ CFU/mL.

The EHP isolate used in this study was obtained from a candidate SPF population of *P*. *vannamei* infected with EHP (Thailand isolate). In order to produce EHP-infected shrimp, a co-habitation challenge was conducted by rearing fifty SPF *P*. *vannamei* (average weight 2.5–3.5 g) with fifty confirmed EHP-positive shrimp in a 1000 L tank for 60 days. The hepatopancreas of the experimentally challenged shrimp were dissected and preserved in Davidson’s fixative and 95% ethanol. EHP infection in the SPF shrimp was confirmed by PCR and histopathology at the end of the cohabitation challenge.

### Experimental challenge to reproduce WFS

To reproduce WFS in a controlled laboratory setting, an experimental bacterial immersion challenge was conducted by using the *V*. *parahaemolyticus* (WFS isolate) as an inoculum and both SPF and EHP-infected population of *P*. *vannamei*. Two independent trials were conducted (Trial 1 and Trail 2). In each trial, four 90-L tanks were filled with artificial seawater adjusted to 30 ppt (Crystal Sea Marinex, Baltimore, Maryland), the temperature was kept at 26°C (±0.6) and the pH ranged from 7.5–8.0. For trial 1, seven SPF and eight EHP-infected *P*. *vannamei* (average weight 6.0. ±1.1 g) were used whereas in trial 2, ten SPF and eight EHP-infected *P*. *vannamei* (average weight: 7.1±2.0 g) were used for the experimental infection. The setup of the tanks for the trials 1 and 2 is summarized in Table 1.

**Table 1:** Experimental design and comparative survival of shrimp pre-infected with EHP and challenged with *V*. *parahaemolyticus* (WFS isolate) in two independent trials.

Trial	Tank	Group	Number of animals (initial)	Number of animals (final)	Final survival	CV%	WFS
1	1	SPF Negative control	7	6	85.7%	21.4	No
	2	SPF +*V*. *parahaemolyticus*	7	6	85.7%	27.6	No
	3	EHP positive control	8	8	100.0%	41.6	No
	4	EHP + *V*. *parahaemolyticus*	7	4	57.1%	40.0	Yes
2	1	SPF Negative control	10	9	90.0%	12.0	No
	2	SPF + *V*. *parahaemolyticus*	10	8	80.0%	10.1	No
	3	EHP positive control	9	4	44.4%	30.8	No
	4	EHP + *V*. *parahaemolyticus*	8	2	25.0%	40.7	Yes

For both trials, *V*. *parahaemolyticus* challenge was conducted via an immersion challenge by adding an overnight culture into the aquaria to obtain a final bacterial concentration of 1.0 × 10^6^ CFU/mL. The bacterial inoculation was conducted four times throughout the challenge on 0, 1, 4, and 18 days post-infection (dpi). The EHP positive control group was not inoculated with *V*. *parahaemolyticus*, while the SPF group was not infected with EHP nor *V*. *parahaemolyticus*. Mortality, presence of white fecal strings and white color in the gastrointestinal tract (GI-Tract) were recorded daily throughout the experiment. Moribund as well as surviving shrimp at the end of the experiment were fixed in Davidson’s AFA fixative [20]. At the termination of the experimental challenge, surviving animals were longitudinally bisected into two halves with one half fixed in Davidson’s (AFA) fixative [20] and the remaining half, was preserved in 95% ethanol for EHP detection by PCR. The duration of the experimental challenge was 23 and 30 days for trials 1 and 2, respectively.

### Histopathology and *in-situ* hybridization

The Davidson’s alcohol-formalin-acetic acid (AFA)-fixed samples were processed, embedded in paraffin, and sectioned (5 μm thick) per standard methods [20, 21]. After staining with hematoxylin and eosin (H&E), the sections were analyzed by light microscopy. The severity of the EHP infection/lesion was graded from G0-G4 according to Lightner [21] with G0 being absent of the disease and G4 being the presence of severe lesions and advanced tissue destruction.

The EHP 510 F (5’-GCC TGA GAG ATG GCT CCC ACG T-3’) and EHP 510 R (5’-GCG TAC TAT CCC CAG AGC CCG A-3’) primers [12] were used for *in situ* hybridization (ISH). These primers were tailed at 3′-end with digoxigenin-11-dUTP (Integrated DNA Technologies®, San Diego, CA). The Davidson’s fixed shrimp were processed, and tissue sections were made (5 μm thick) (Lightner 1996a). After deparaffinization, hydration, proteinase K digestion, and pre-hybridization, sections were overlaid with 100 μL hybridization solution containing DIG-labeled primers (100 fmol). The slides were placed on a heated surface at 90°C for 10 min and hybridized overnight at 50°C. Final detection was performed with an anti-digoxigenin antibody conjugated to alkaline phosphatase (Roche) that was visualized using nitro blue tetrazolium and 5-bromo-4- chloro-3-indolyl phosphate [12].

### PCR and quantitative PCR for detection of *V*. *parahaemolyticus* and quantification of EHP

Detection of *V*. *parahaemolyticus* was carried out by end-point PCR using *toxR* as the target gene, as previously published [19]. A 368-bp fragment was amplified using the primers, F: 5′-GTC-TTC-TGA-CGC-AAT-CGT-TG-3′ and R: 5′-ATA-CGA-GTG-GTT-GCT-GTC-ATG-3′. PuReTaq Ready-to-Go PCR beads (GE Healthcare) were used for amplification using the following cycling parameters: initial denaturation at 94°C for 3 min, followed by 35 cycles of 94°C for 30s, 63°C for 30s, and 72°C for 30s, and a final extension at 72°C for 5 min. Following amplification, the PCR products were electrophoresed in 1.5% agarose gels containing 1X GelRed and visualized under ultraviolet light and digitally photographed by the Gel documentation system from Bio-Rad ®. EHP detection and quantification was carried out by quantitative PCR (qPCR) based on the EHP SSU rDNA following a published method [22]. Primer F:157 (5′-AGT AAA CTA TGC CGA CAA-3′) and primer R:157 (5′-T TAA GCA GCA CAA TCC-3′), and a TaqMan probe (5-FAM-TCC TGG TAG TGT CCT TCC GT-TAMRA-3′) were used. DNA concentration was normalized at 20 ng ul^-1^. The amplification reactions were conducted as follows: 0.5 μM of each primer, 0.1 μM of TaqMan probe, 1× TaqMan Fast Virus 1-Step Master Mix (Life Technologies), 50 ng of DNA and HPLC water in a reaction volume of 10 μl. The qPCR profile consisted of 20s at 95°C followed by 40 cycles of 1s at 95°C and 20s at 60°C. Amplification detection and data analysis for qPCR assays were carried out with the StepOnePlus real-time PCR system (Life Technologies).

### Statistical analysis

Statistical analyses were conducted using GraphPad Prism Version 6.0. One-way ANOVA (α = 0.05) was used to determine statistical variances between groups. Tukey’s multiple comparisons post-hoc was used to analyze statistical difference between the EHP load among shrimp sampled from different treatments after logarithmic transformation of data.

## Results

### Identification of *Vibrio sp*. isolate from *Penaeus vannamei* shrimp displaying WFS

**Fig 1:**
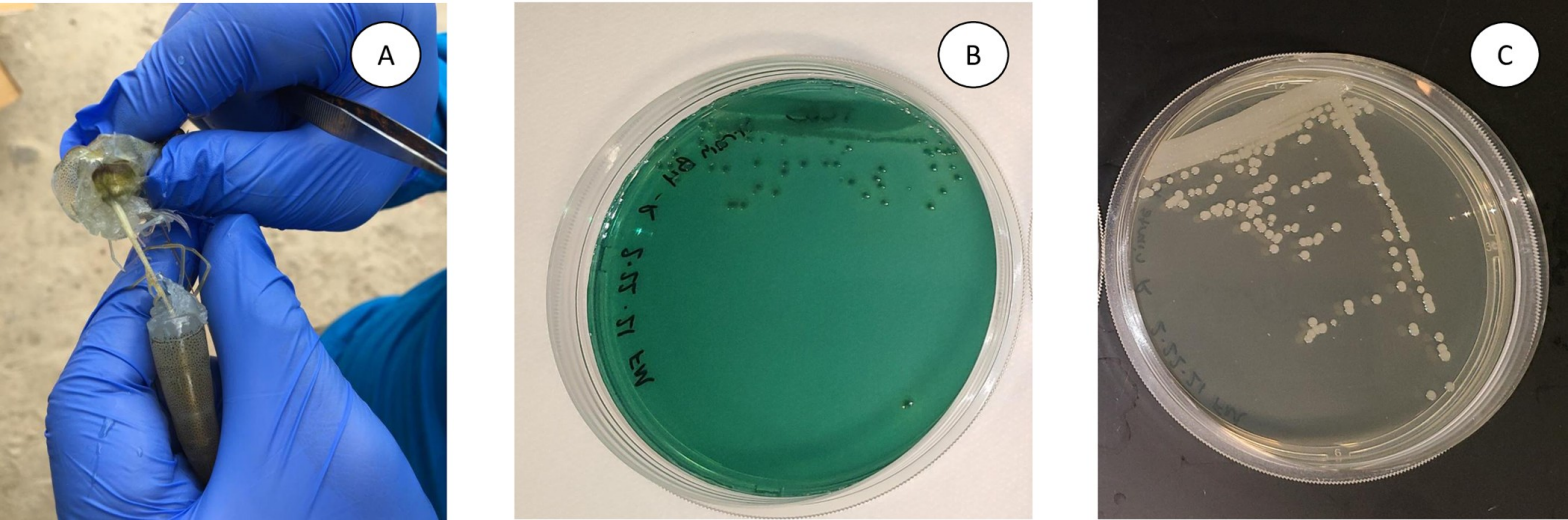
Isolation of *Vibrio parahaemolyticus* from *Penaeus vannamei* Gi-tract displaying WFS. A juvenile *P*. *vannamei* shrimp displaying white gastrointestinal (GI) tract, a clinical manifestation of WFS. A section of the GI tract was plated on a TCBS plate (a). CFU Sucrose negative indicating *Vibrio parahaemolyticus* growth in TCBS plate (b). Growth of *V*. *parahaemolyticus* in TSA+2.5% NaCl medium (c).

**Fig 2:**
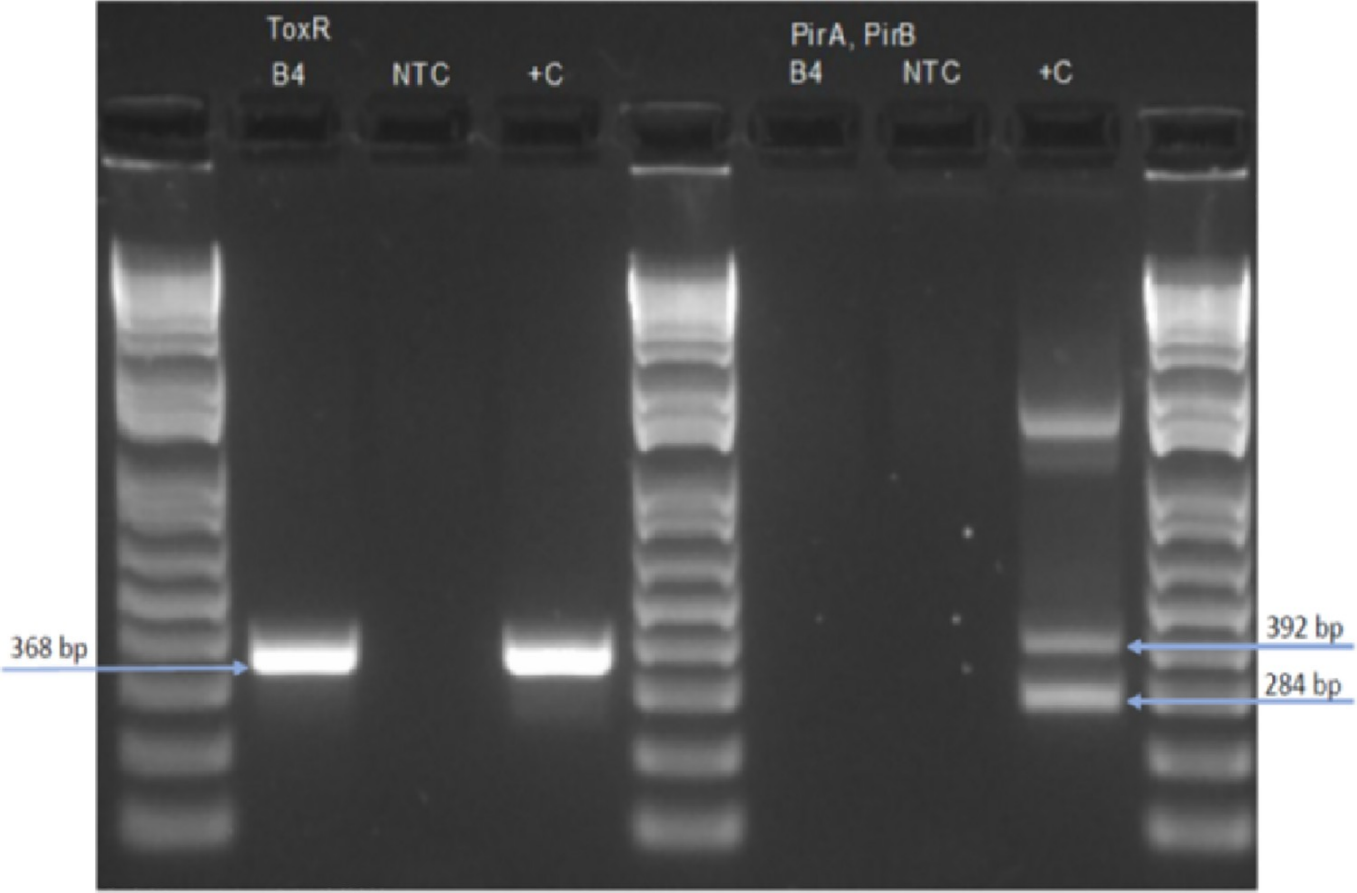
The PCR detection of *toxR* and PirAB^*vp*^ genes in *Vibrio parahaemolyticus* isolated from *Penaeus vannamei* shrimp displaying WFS. The *toxR* gene was amplified from a *Vibrio parahaemolyticus* CFU (B4) (left panel) but the binary toxin genes, PirA^*vp*^ and PirB^*vp*^ could not be detected in B4 (right panel). Column M:1 kb plus ladder molecular weight marker, B4: *V*. *parahaemolyticus* isolate. NTC: no template control; +C left: *V*. *parahaemolyticus* positive control. +C right: *VP*_AHPND_ positive control.

A loopful of the GI tract of a *P*. *vannamei* shrimp displaying clinical manifestation of WFS was plated on a TCBS plate. Presence of only CFU Sucrose negative on TCBS plate (green colonies) (Fig 1) indicate that the bacterial isolate most likely represents *V*. *parahaemolyticus*. When plated on TSA+ plate, white CFU were observed. The 16S rRNA gene (1455 bp) was successfully amplified from an isolated CFU and the amplicon sequenced using forward and the reverse primers used for the PCR amplification. The consensus sequence showed highest identity with several strains of *V*. *parahaemolyticus* (>99% identity) in NCBI database (GenBank accession number: MT534026.1, MT534020.1, MT534018.1, MT534017.1, and CP051111.1). The *V*. *parahaemolyticus* 16SrRNA sequence was deposited in GenBank with the accession numbers MW526256. Interestingly, NCBI entries showing highest identity were also isolated from shrimp farms as well. To further confirm the identity of *Vibrio* isolate, a 368 bp DNA representing the *toxR* gene was amplified by PCR (Fig 2). However, the PirAB^*vp*^ gene could not be amplified from the same isolate (Fig 2) indicating the bacterial isolate represent a non-acute hepatopancreatic necrosis disease (AHPND)-causing *V*. *parahaemolyticus*.

### Experimental challenge of EHP-infected shrimp with *Vibrio parahaemolyticus*

**Fig 3:**
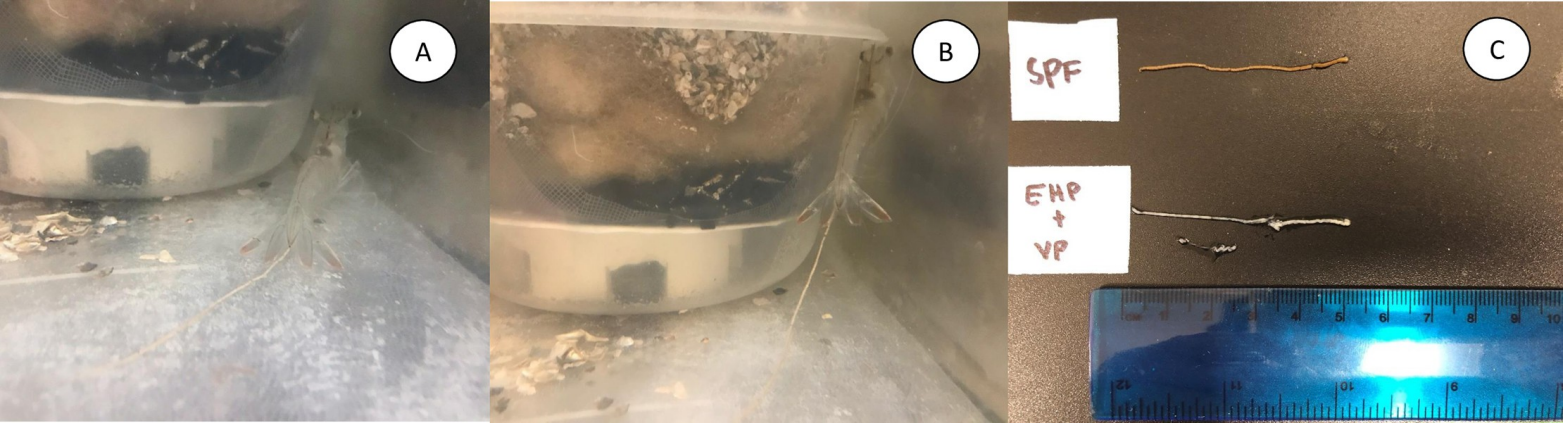
Successful reproduction of WFS in *Penaeus vannamei* under a laboratory experimental condition. Shrimp displaying clinical signs of White Feces Syndrome (WFS) in shrimp that were pre-infected with EHP and challenged with *Vibrio parahaemolyticus* via immersion route. White to yellowish colored fecal strings are observed in animals in different treatments. The black arrow indicates white fecal string adhering to the anal opening in EHP infected shrimp that were challenged with *V*. *parahaemolyticus*.

The EHP-infected shrimp were challenged with *V*. *parahaemolyticus* via immersion. In both trials 1 and 2, the white discoloration of the GI tract in shrimp was observed initially at 8 dpi and only in the tank with shrimp pre-infected with EHP. Subsequently, white fecal strings adhering to the anal opening and fecal string floating on tank water were observed for this treatment (Fig 3). However, no shrimp either infected with EHP alone or SPF shrimp challenged with *V*. *parahaemolyticus* display any clinical signs of WFS. In the electronic supplementary material, there is one video showing shrimp displaying WFS (S1 Video). The final survival at the termination of the experiment in both trials 1 and 2 was lower in the treatment, EHP+*V*. *parahaemolyticus* compared to the other treatments (Table 1). The body weight was recorded for each surviving shrimp in all treatments at the time of termination of the experiment. In both trials, the coefficient of variation (CV%) was high (~40%) in the treatment where shrimp were infected with EHP.

### H&E and *in-situ* hybridization

**Fig 4:**
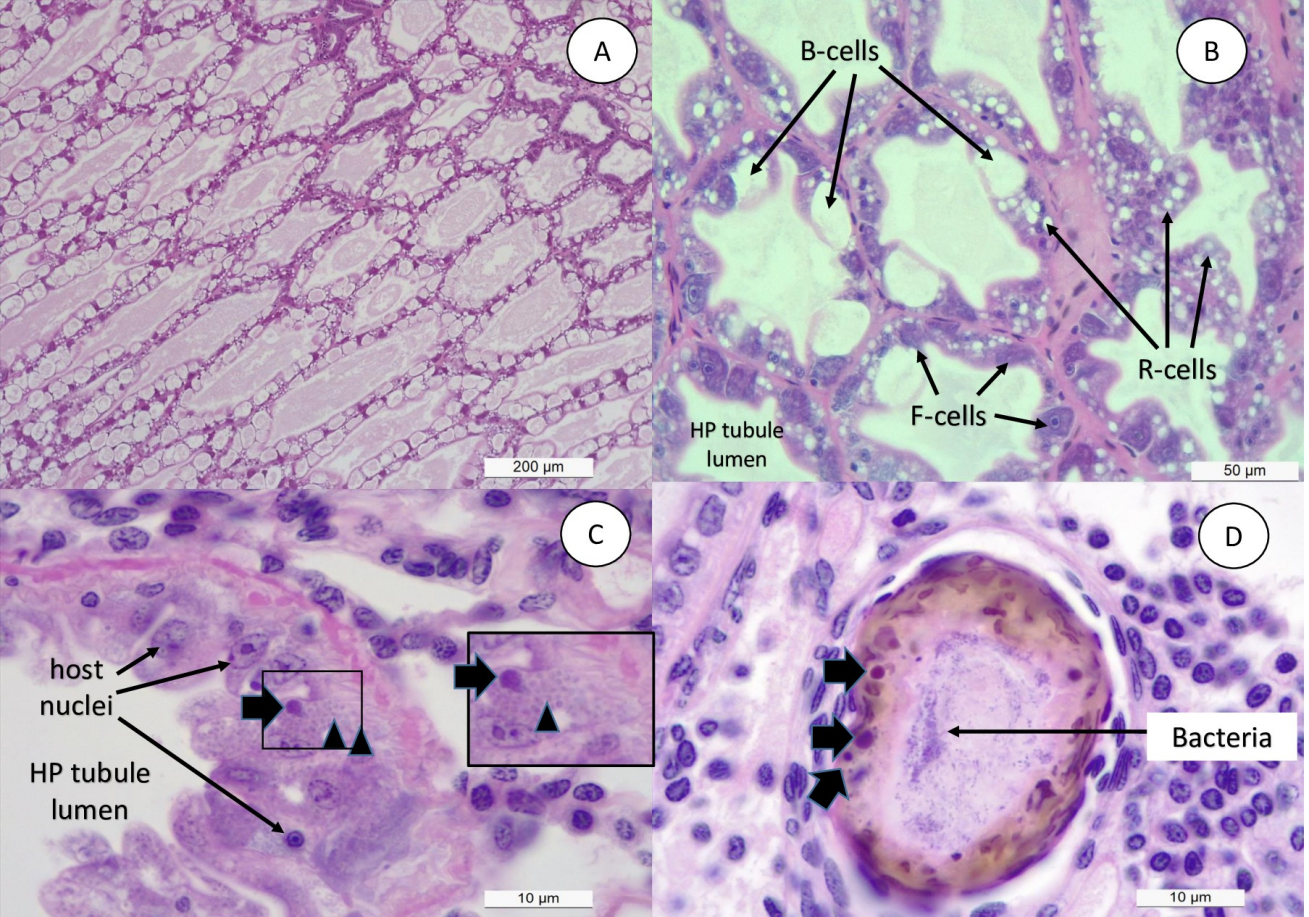
Histopathology analysis of hepatopancreas tissue. H&E (Mayer–Bennet hematoxylin and eosin-phloxine) staining of hepatopancreas tissue of *Penaeus vannamei* from four experimental groups: SPF negative control (A), SPF+*V*. *parahaemolyticus* (B), EHP positive control (C), and EHP+*V*. *parahaemolyticus* (D). Presence of cytoplasmic inclusion bodies (black arrows) that correspond to a plasmodium stage and spores (arrowhead) typical of EHP infection are shown. The scale bars of 200 μm (A), 50 μm (B) and 10 μm (C-D) are indicated.

**Fig 5:**
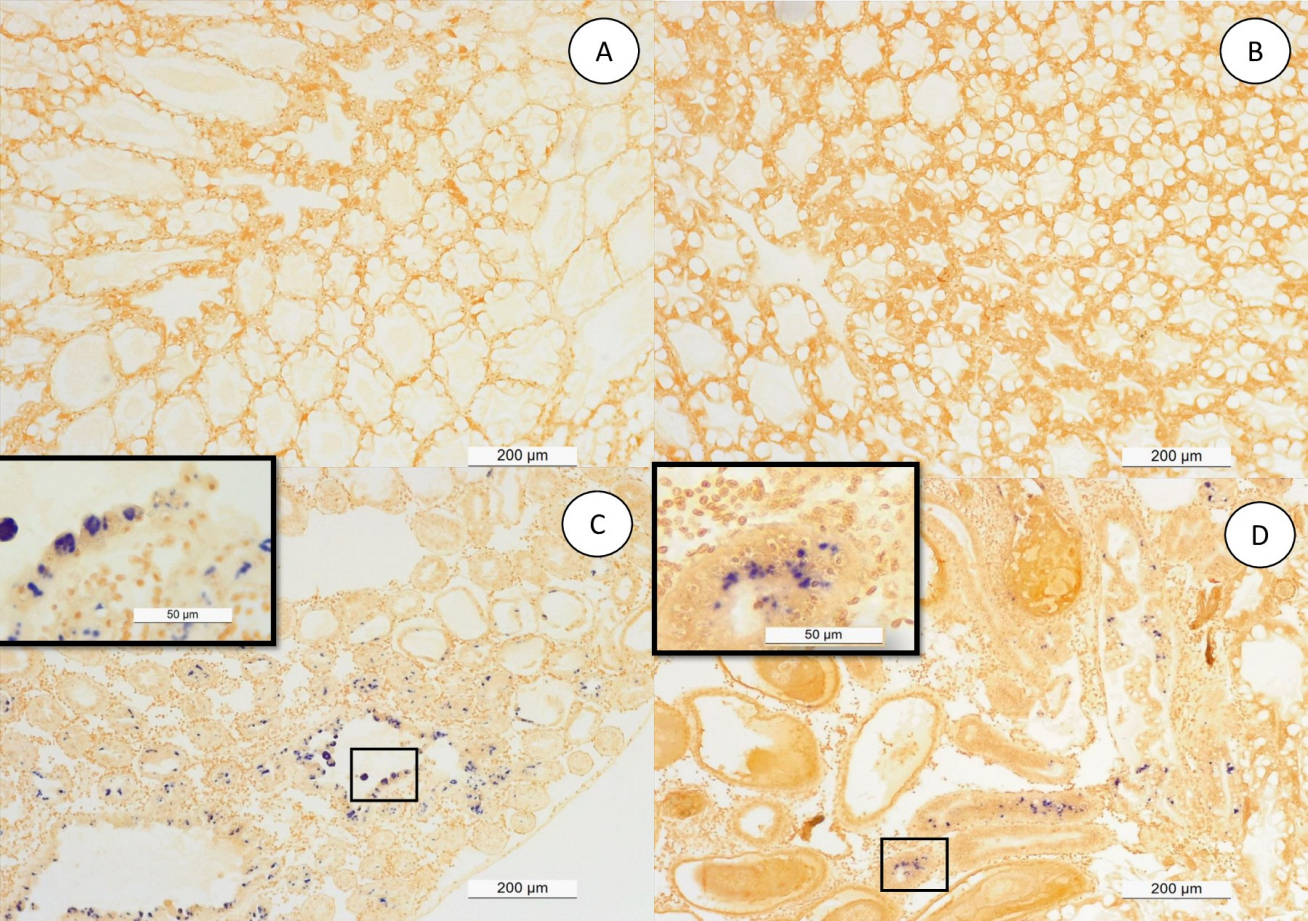
*In-situ* hybridization with digoxigenin-labeled *EHP* probes. Hepatopancreas tissues section parallel to a section used for H&E staining (Fig 4) on four experimental groups: SPF negative control (A), SPF+*V*. *parahaemolyticus* (B), EHP positive control (C), and EHP+*V*. *parahaemolyticus* (D). The presence of dark blue/purple precipitates indicates the presence of EHP. A scale bar of 200 μm (A-B) and 50 μm (C-D) are indicated.

Shrimp samples collected from different treatments in trial 1 & 2 were analyzed by histopathology, *in-situ* hybridization using EHP-specific probe, and real-time PCR. The summary of the results are presented in Table 2. No EHP could be detected in shrimp sampled from the negative control and SPF+*V*. *parahaemolyticus* challenged tanks. However, in both trials, EHP was detected in all the animals in the EHP only (positive control) as well as in EHP + *V*. *parahaemolyticus* challenged group. For confirmation of EHP infection, samples from trial 1 were further analyzed by *in-situ* hybridization using EHP-specific probe. The presence of plasmodium and spore stages typical of EHP infection are shown in Fig 4C and 4D. Black arrows show typical regular and irregular plasmodium stages of EHP, whereas the arrow heads indicate the presence of EHP spores. *In situ* hybridization using EHP-specific DIG-labeled 18S rRNA probe reacted intensely to the inclusion bodies within the cytoplasm of the infected cells, thus confirming the presence of EHP. The severity of infection was graded G1 to G4 (Fig 5C and 5D). SHPN was present in shrimp collected from the EHP positive control and EHP+ *V*. *parahaemolyticus* treatments in both trials (Table 2). The presence of EHP and bacteria within the same HP tubule is shown in Fig 4D. No other histological lesions were found in any of the shrimp analyzed.

**Table 2:** A summary of histopathology and PCR data of *Penaeus vannamei* pre-infected with EHP and challenged with *Vibrio* sp.

Trial	Treatments	Clinical signs	Number of samples	EHP & SHPN detection by H&E histology		EHP detection by *in situ* hybridization	EHP detection by real time-PCR
				EHP (%)	SHPN (%)		
**1**	SPF Negative control	No WFS	6	0	0	0%	0%
	SPF+*V*. *parahaemolyticus*.	No WFS	6	0	0	0%	0%
	EHP positive control	No WFS	8	100	37.5%	100%	100%
	EHP+ *V*. *parahaemolyticus*.	WFS	6	100	83.3%	100%	100%
**2**	SPF Negative control	No WFS	6	0	0%	NT	0%
	SPF+ *V*. *parahaemolyticus*	No WFS	6	0	0%	NT	0%
	EHP positive control	No WFS	5	100	60%	NT	100%
	EHP+ *V*. *parahaemolyticus*.	WFS	5	100	60%	NT	100%

The presence or absence of clinical signs, H&E histology, *in-situ* hybridization using EHP-specific probe, and real-time PCR results are summarized. SHPN: Septic hepatopancreatic necrosis. NT = Not tested.

### Detection and quantification of *EHP* by real-time PCR

**Fig 6:**
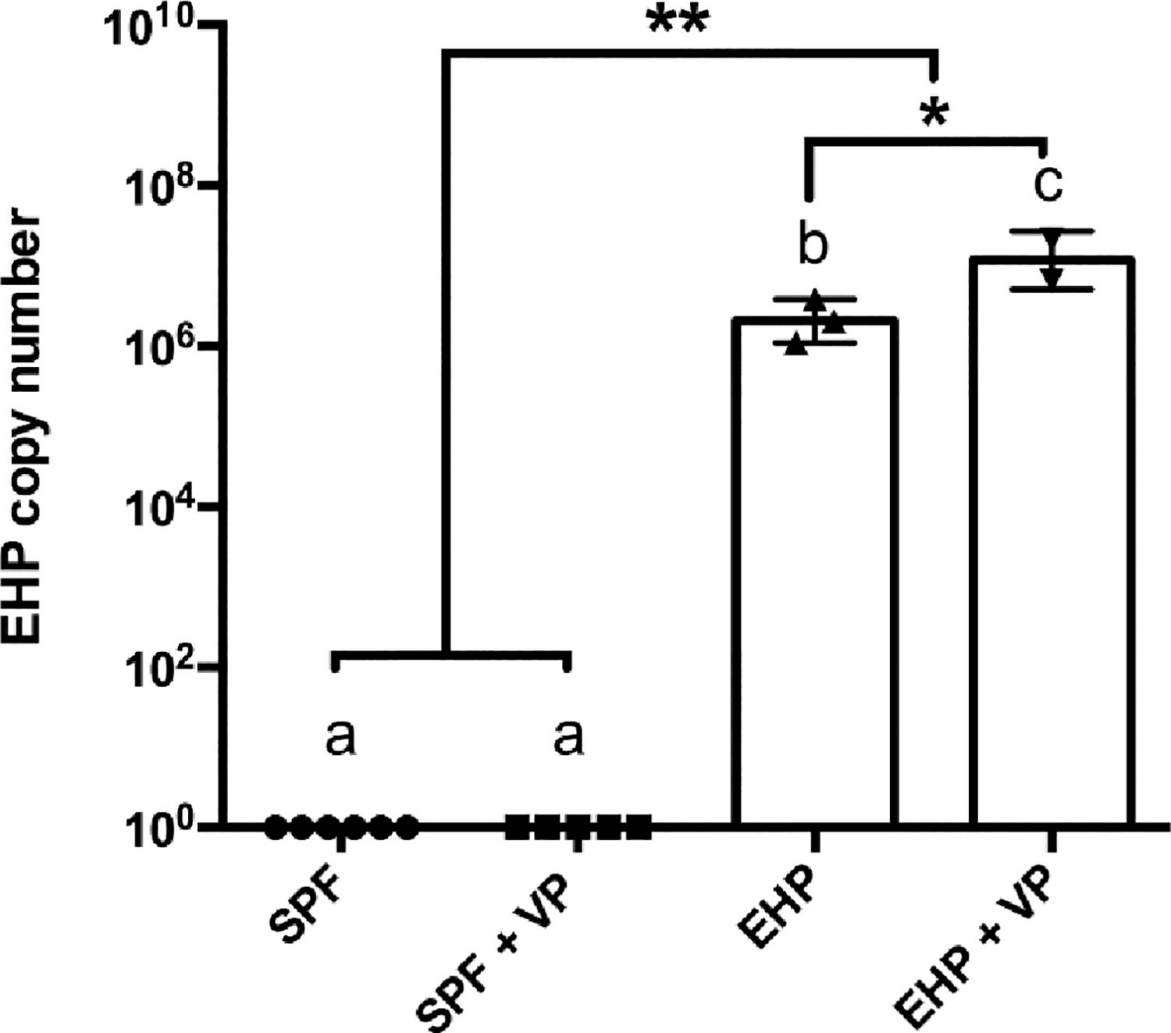
EHP copy number in the experimentally challenged *Penaeus vannamei* shrimp. Samples of hepatopancreas (HP) were analyzed for EHP load by qPCR. EHP copy number is represented as the Mean ±SE. One-way ANOVA with Tukey’s multiple comparisons post-hoc was used to analyze statistical difference. Letters (a-c) indicate significant differences between groups and asterisk “*” indicate significant differences at *P value* < 0.05 and “**” at *P value* < 0.01.

Samples of the hepatopancreas from the four different groups were analyzed by real-time PCR to determine the EHP load in trial 1 and 2. All samples from the group EHP and EHP+*V*. *parahaemolyticus*. were positive for EHP (Table 2). As expected EHP was not detected in the shrimp from the negative control and animals challenged with *V*. *parahaemolyticus* alone. The parasite load within the EHP positive control group ranged between 1.0 × 10^6^ and 3.8 × 10^6^ copiesng^-1^ of DNA, with a mean value of 2.3 × 10^6^ copies ng^-1^. The corresponding parasite load in the EHP+*V*. *parahaemolyticus* treatment ranged from 6.5 × 10^6^ to 2.1 × 10^7^ with a geometric mean of 1.4 × 10^7^ copiesng^-1^ of DNA (Fig 6).

## Discussion

Here we describe for the first time the experimental reproduction of WFS in shrimp, *P*. *vannamei* pre-infected with EHP (primary pathogen) and challenged with a particular *Vibrio parahaemolyticus* (secondary pathogen) isolated from the GI tract of a shrimp displaying WFS. The data presented here provide empirical support of a strong association of EHP with WFS as recorded in different regions of the world [6, 8, 10]. In Indonesia during 2016–2018, and in Venezuela in 2019, we observed shrimp with clinical signs of WFS displayed histological lesions typical of EHP and SHPN. The histological findings presented here revealed a similar scenario as shrimp experiencing clinical manifestations of WFS under laboratory challenged conditions also displayed histological lesions that are reminiscent of EHP and SHPN pathology.

White Feces Syndrome has proven to have one of the most elusive etiology of any of the shrimp disease that have been characterized to date. It has been more than a decade since the first formal report of EHP infected shrimp showing WFS was published [4], although anecdotal evidence suggests WFS has been observed in shrimp grow out pond prior to this publication. Despite the presence of WFS over a decade, the etiology of the syndrome remains inconclusive. There are also several publications with contradictory claims with respect to the role of EHP in WFS. For example, some authors reported WFS is not caused by EHP [14] while others found a strong evidence of a clear link between EHP and WFS [8]. There is one report claiming *Vibrio cholerae* as the etiologic agent of WFS [23] although this finding has not been confirmed by other researchers from anywhere else despite the fact WFS is an emergent problem worldwide. Several other studies aimed to investigate the WFS phenomenon have also found some association between WFS and different etiologies. Fecal strings containing vermiform structures called aggregated transformed microvilli (ATM) has been reported as one of the causative agents of WFS in Thailand [24]. WFS appears to be correlated with coinfection with EHP and other opportunistic *Vibrio* spp. that cause SHPN [6, 10]. In India, running mortality syndrome (RMS) has been reported in shrimp displaying WFS and SHPN [25]. Researchers studying microbiome in WFS affected shrimp reported a significant increase in *Candidatus Bacilloplasma and Phascolarctobacterium* and decreases in *Paracoccus* and *Lactococcus* may contribute to WFS in shrimp [26]. Heavy gregarine infestation, vibriosis and hemocytic enteritis, some specific bacterial taxon or ‘pathobiome’, and different environmental condition in a grow out pond have been proposed to be the contributory factors for causing WFS [13, 27].

While studies to reproduces WFS in a laboratory setting have been attempted by several authors few have been successful in consistently reproducing this syndrome and determining the etiology. Huang et al. (2020) [28] recently found that transplantation of the intestinal microbiota of shrimp displaying WFS to healthy shrimp leads to similar clinical manifestations as in WFS. They proposed WFS is caused by dysbiosis in intestinal microbiota and this complex disease could be caused by particular pathogens. While a non-infectious etiology [28] is worth further investigation, we cannot disregard the fact that WFS has been consistently associated with particular pathogens notably EHP. Furthermore, the constant co-occurrence of certain patterns of the bacterial communities [27, 28] in shrimp that present WFS lead us to hypothesize that WFS is the result of the synergetic effects of two pathogens with EHP possibly acting as a primary pathogen and an uncharacterized bacteria belonging to the family Vibrionaceae acting as a secondary pathogen.

Under laboratory challenge experiments, we were able to confirm the presence of white fecal strings only in shrimp that were pre-infected with EHP and subsequently exposed to the *Vibrio parahaemolyticus* isolate. This demonstrates that, in order to induce clinical manifestations of WFS, both EHP and this particular *Vibrio parahaemolyticus* isolate are required. Shrimp exposed only to EHP or *Vibrio parahaemolyticus* did not develop WFS. Therefore, WFS manifestation involves a unique combination of two pathogens that had not been observed previously for any infectious disease of shrimp known to date. Typically, in a shrimp grow out pond, the cultured population starts showing the presence of EHP first. Later on, during the grow-out cycle, shrimp start developing white discoloration of the GI tract, and floating white fecal strings start appearing in the pond. By H&E histology, we observed a similar sequential order in cellular pathology during the infection process. A primary pathogen, in this case EHP, caused the typical histological lesions and later on affected shrimp display SHPN caused by opportunistic *V*. *parahaemolyticus* that was used to challenge the EHP-infected shrimp. The histopathology image in Fig 4D clearly displays co-infection of EHP as represented by a typical plasmodium inclusion and bacteria in the same HP tubule. This establishes the role of a primary pathogen like EHP to accentuate the impact of opportunistic bacteria like *Vibrio sp*. resulting WFS.

Shrimp in the treatment group EHP+*V*. *parahaemolyticus* in both trials, displayed a high size disparity which is higher or comparable to shrimp infected with EHP or *V*. *parahaemolyticus* alone (Table 1). A similar finding was also reported by Liu et al. (2015)[29]. These authors reported the impact of EHP on size variation of shrimp in a grow-out farm when the pathogen load was found to be >10^3^ copies/ng total DNA in hepatopancreatic tissue. In the current study, the mean EHP load in shrimp displaying clinical signs of WFS was 1.4 × 10^7^ copies ng^-1^ of extracted DNA, which is higher from what Liu et al. (2015) mentioned is required to observe high size variation. In previous studies, shrimp displaying WFS show a copy number in the order of ~ 10^6^ copies ng^-1^ of extracted HP DNA [8].

The reproduction of WFS in shrimp infected with EHP and challenged with *V*. *parahaemolyticus* is very likely due to the synergistic actions of the microsporidian and a particular bacterial strain on hepatopancreatic tubules. As mentioned previously, Huang and colleagues suggested that WFS is caused by intestinal microbiota dysbiosis [28] and this proposition bears some similarities to our finding. We observed during the early stage of EHP infection in shrimp displaying WFS includes high lipid contents in the hepatopancreatic tubule but some HP cells including the multivacuolated lipid and glycogen storage R-cells and the secretory B-cells being heavily infected. However, at this point, there is no obvious cellular shrimp immune response such as inflammation or hemocytic congestion. Later, as the infection progresses, EHP-infected epithelial cells in the HP tubule slough exposing the underlying basal membrane of the tubule to opportunistic bacteria that rapidly colonizes the affected tubules. Bacterial colonization leads to a cell response of the host resulting in hemocytic inflammation, subsequent melanization and further sloughing. There is a moderate to severe atrophy of the hepatopancreas tubule cells leading to a partial or total dysfunction of the hepatopancreas. In a healthy shrimp, the bulk of the feces generated upon digestion of feed consists of residual vacuoles from the B cells which constitute a major process for packaging and removing waste products from the digestive system [30]. In EHP-infected shrimp, when the cluster of HP cells are released from the tubule to the GI tract, the combination of lipids, digested/undigested feed, EHP, and the bacterial mass will contribute with the formation of a whitish fecal string. It is possible due to the presence of lipid in the feces, the fecal mass becomes lighter compared to feces from a healthy shrimp and floats on the surface of water instead of sinking at the bottom of the pond.

Alterations involving the gastrointestinal track, where a sign of the disease are whitish stools, are common to all animals including humans. In humans, whitish stools are caused by changes in the gut microbiome [31] or by particular pathogens such as *Cryptosporidiu*m spp. [32]. This might also be the case for shrimp WFS, where the disease and therefore the particular signs are caused by two distinct etiologies. Our study clearly shows that the two pathogens induce white feces in shrimp and decisively shows that EHP plays a central role in the development of this syndrome as our research group proposed in previous research.

Our study provides conclusive evidence that EHP is not the only pathogen associated with the presence of WFS. In Thailand, some studies have shown the association of WFS in shrimp population with an increase of *Vibrio* spp. where the intestine in shrimp, with and without WFS, were analyzed. An increase of intestinal *Vibrio* population from a normal shrimp with 1.8x10^7^ CFU/g population versus WFS-shrimp with 6.1x10^7^ CFU/g. Some of these *Vibrio*. spp found in those samples were *V*. *parahaemolyticus*, *V*. *vulnificus*, and *V*. *damsela*. [33]. This corroborates our identification of a strain of *V*. *parahaemolyticus* as the unidentified pathogen co-infecting with EHP on shrimp manifesting WFS. However, it is possible that other *Vibrio* species in addition to *V*. *parahaemolyticus* could induce WFS.

## Conclusions

EHP as a primary enteric pathogen of penaeid shrimp. Infection with EHP in association with a particular strain of *Vibrio parahaemolyticus* leads to the development of WFS. These findings also corroborate and provide an explanation as to why WFS is manifested in areas where EHP and *V*. *parahaemolyticus* are also endemic.

## Supporting information

S1 Raw images (ZIP)

S2 Raw images (TIF)

S3 Raw images (ZIP)

S4 Raw images (ZIP)

S5 Raw images (ZIP)

S1 Video Video of a whiteleg shrimp *Penaeus vannamei* from an experimental infection. This specimen was pre-infected with EHP and then challenge with a strain of *Vibrio parahaemolyticus*. The white fecal strings still attached to the anal cavity is observed.
(MOV)
