# Supplementary figures and images for: Experimental reproduction of White Feces Syndrome in whiteleg shrimp, Penaeus vannamei
Source: PLoS One. 2021 Dec 23;16(12):e0261289. doi: 10.1371/journal.pone.0261289 (PMC8699691; doi:10.1371/journal.pone.0261289)

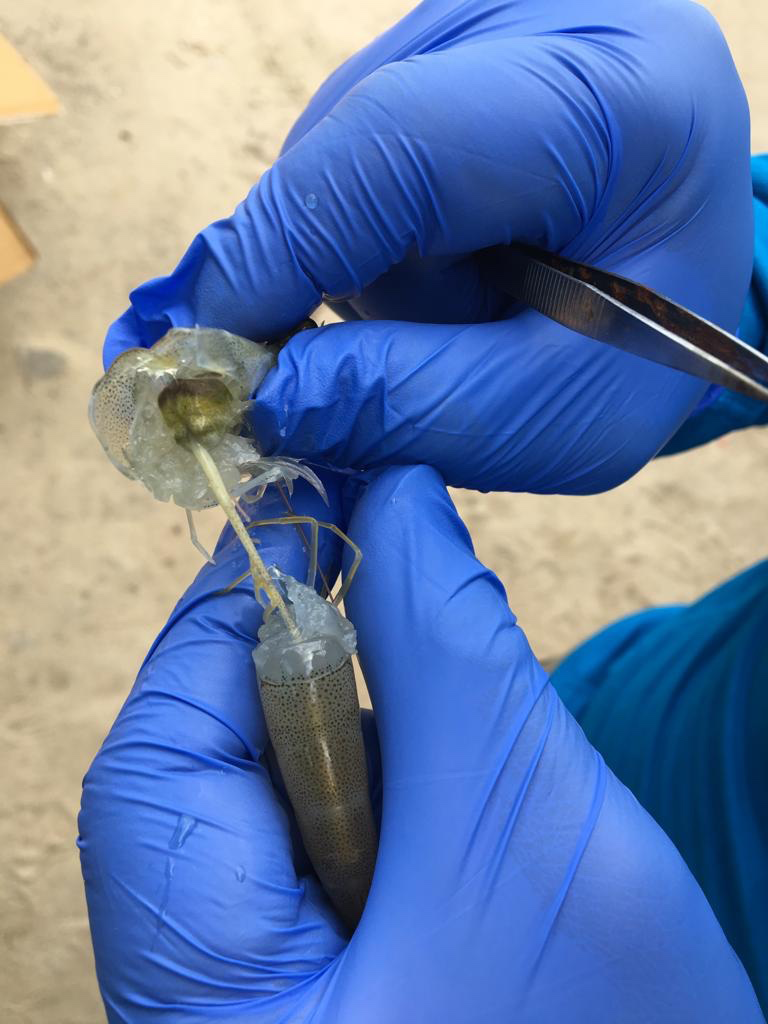

Supplement: S1 Raw images — (ZIP) [file pone.0261289.s001.zip › Fig 1A raw.tif]

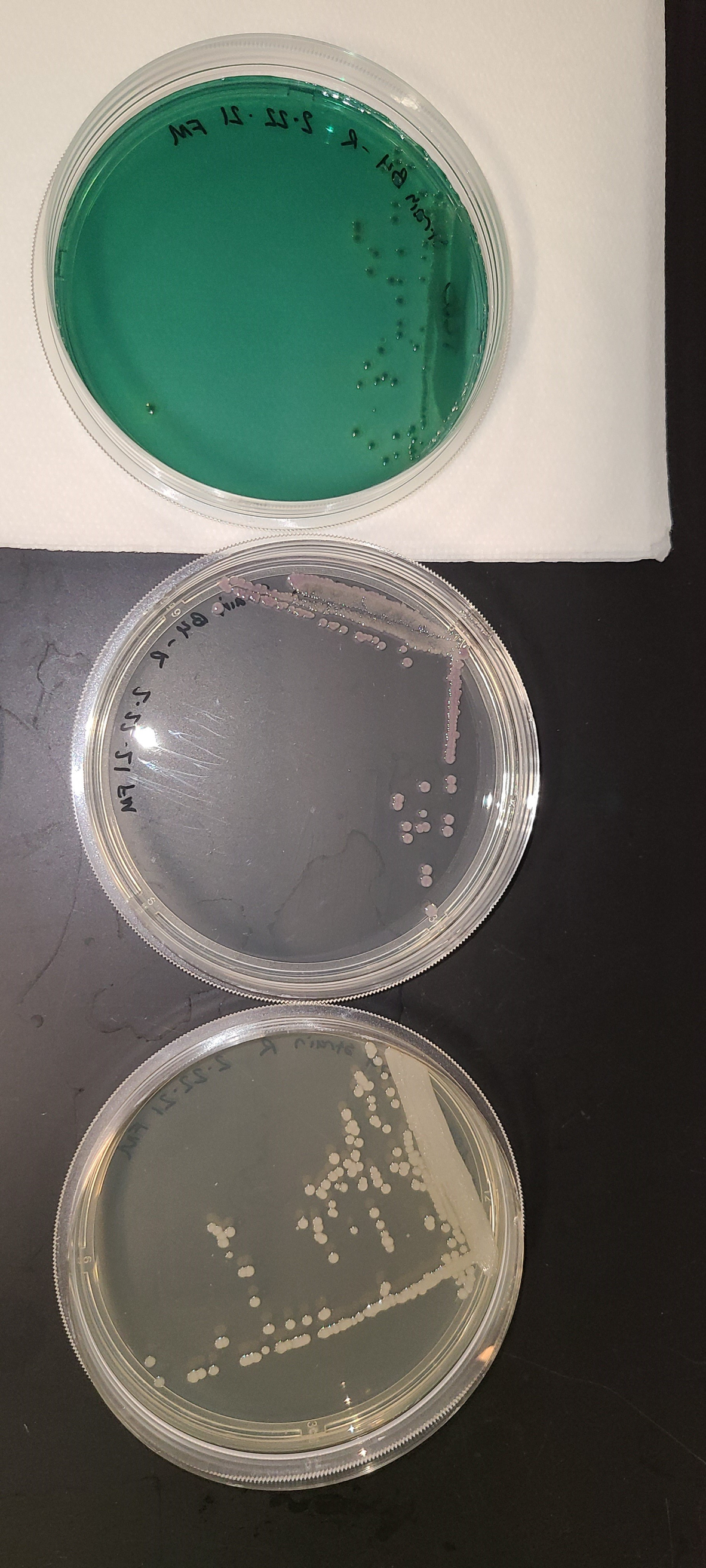

Supplement: S1 Raw images — (ZIP) [file pone.0261289.s001.zip › Fig 1B & C raw.tif]

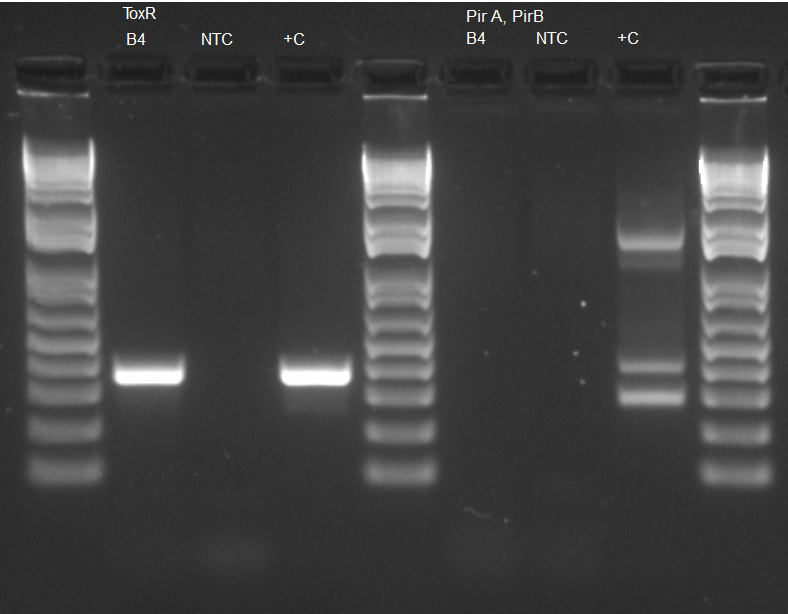

Supplement: S2 Raw images — (TIF) [file pone.0261289.s002.tif]

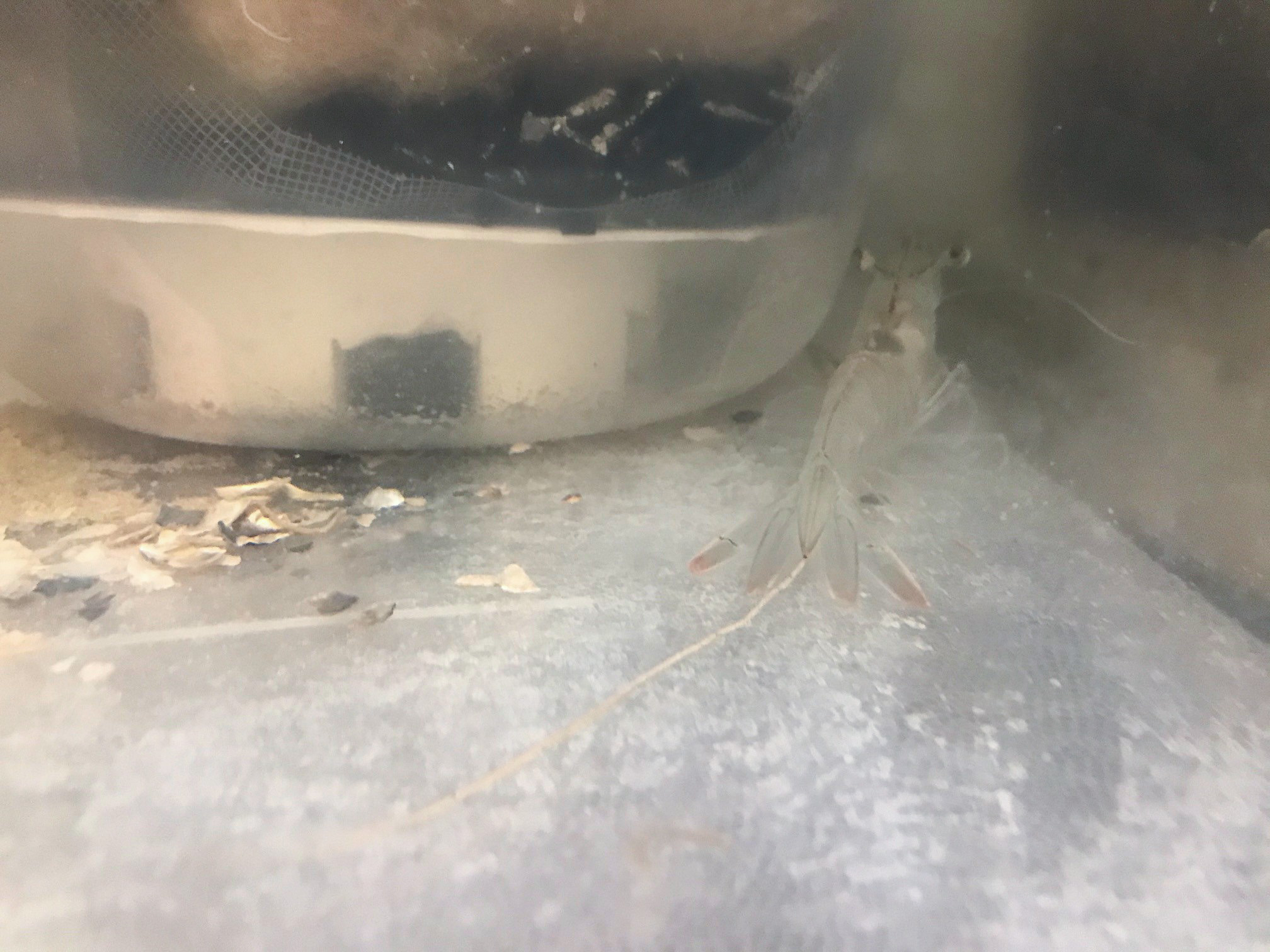

Supplement: S3 Raw images — (ZIP) [file pone.0261289.s003.zip › Fig 3A raw.tif]

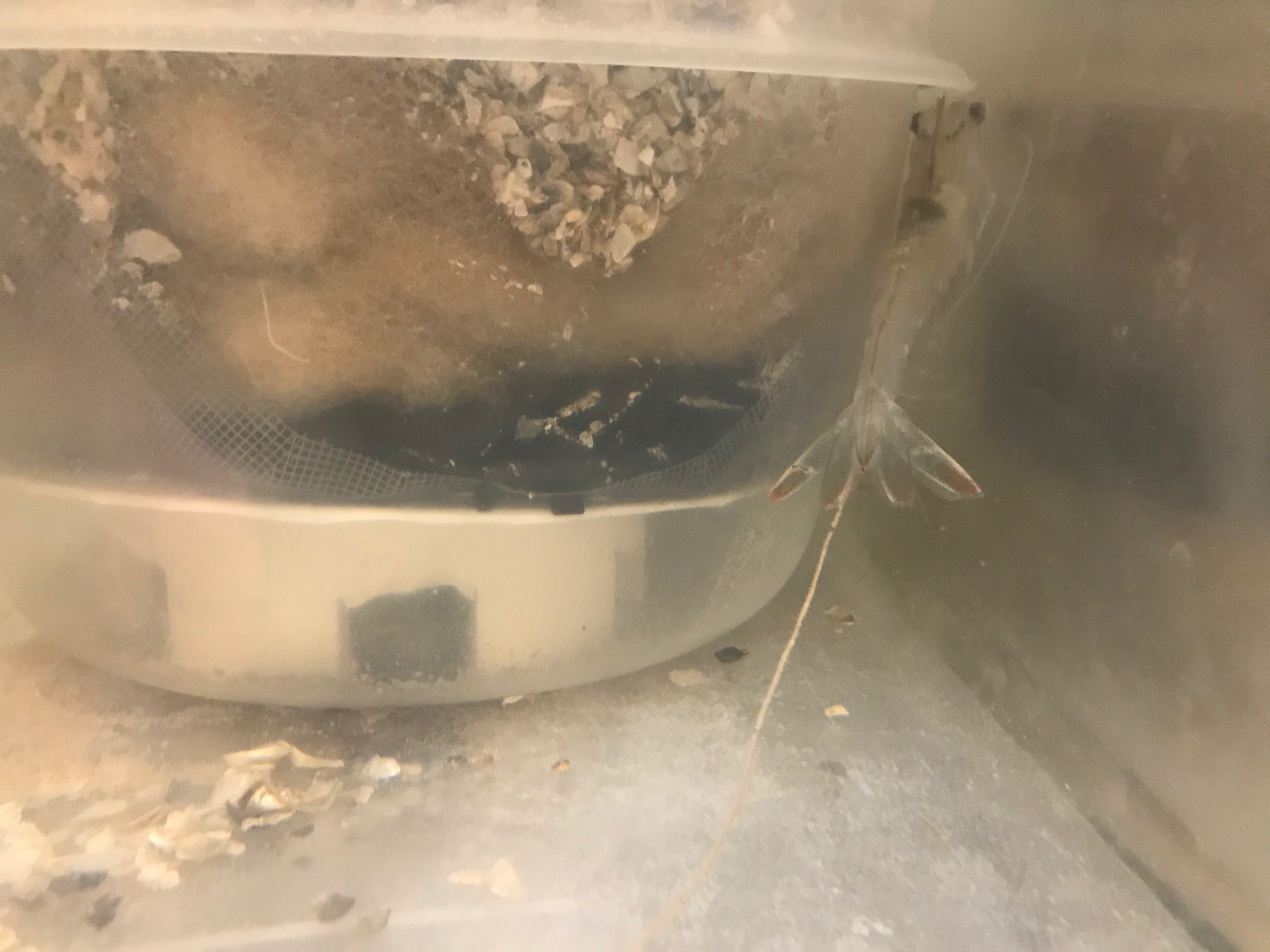

Supplement: S3 Raw images — (ZIP) [file pone.0261289.s003.zip › Fig 3B raw.tif]

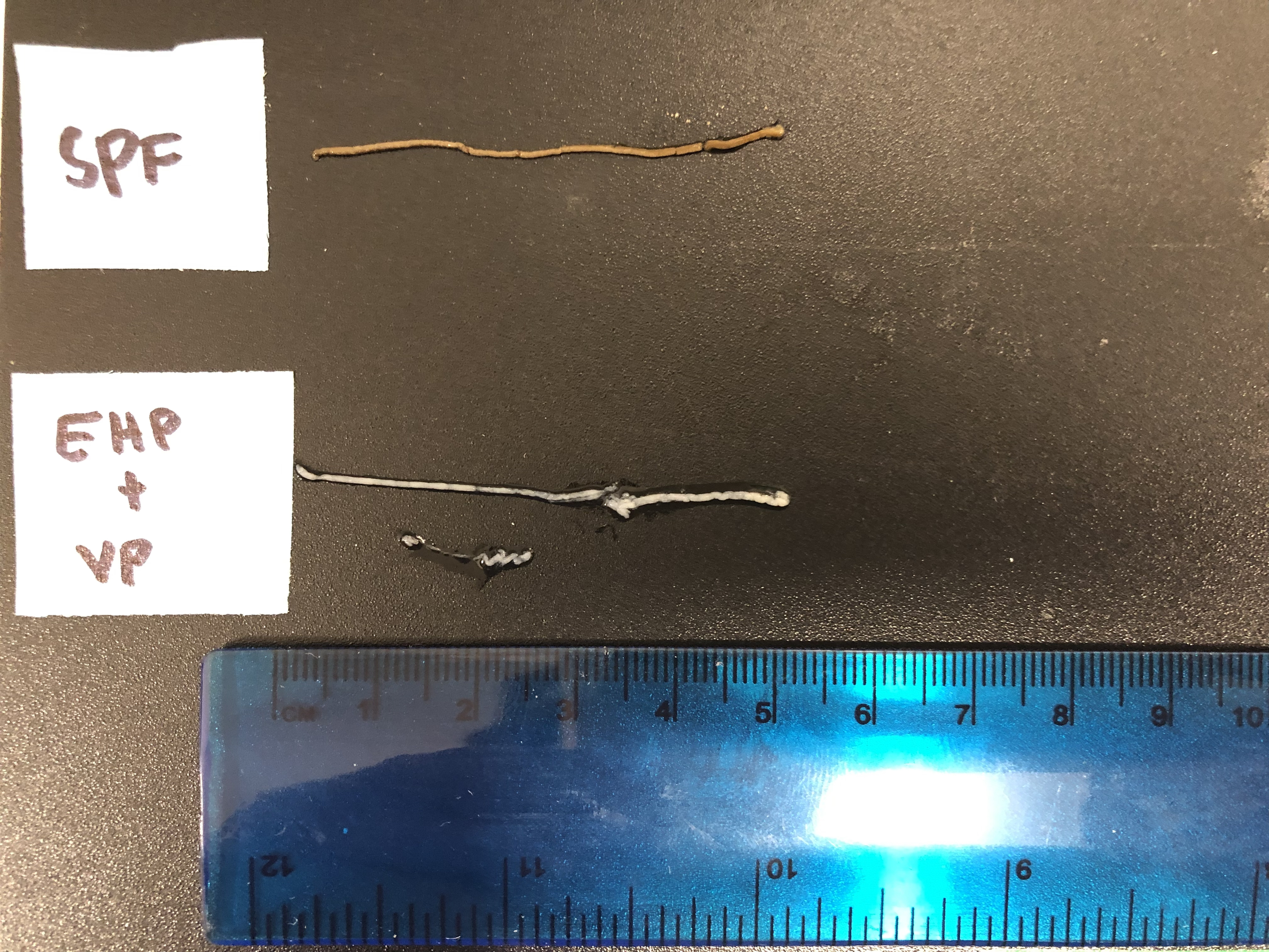

Supplement: S3 Raw images — (ZIP) [file pone.0261289.s003.zip › Fig 3C raw.tif]

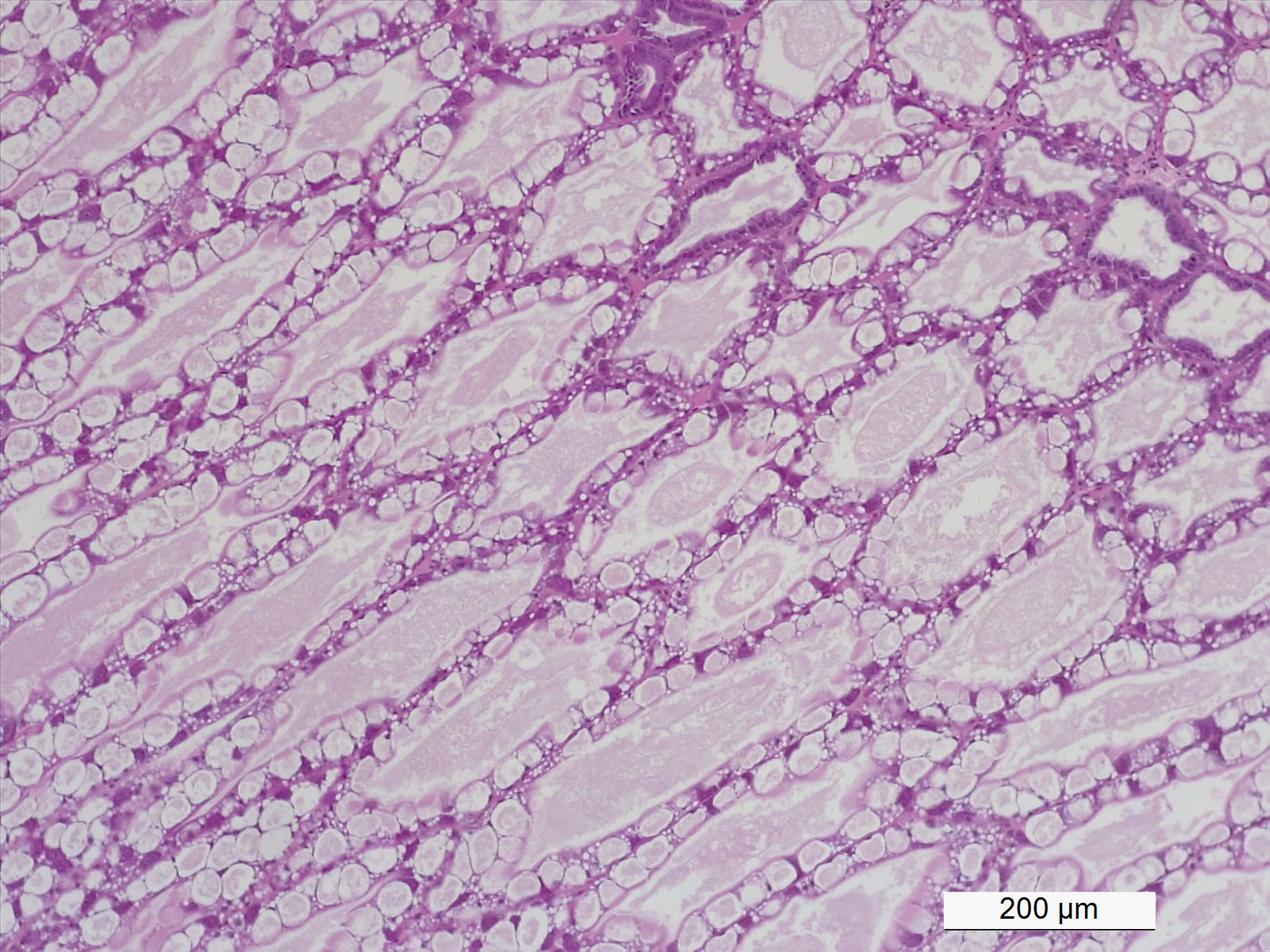

Supplement: S4 Raw images — (ZIP) [file pone.0261289.s004.zip › Fig 4A raw.tif]

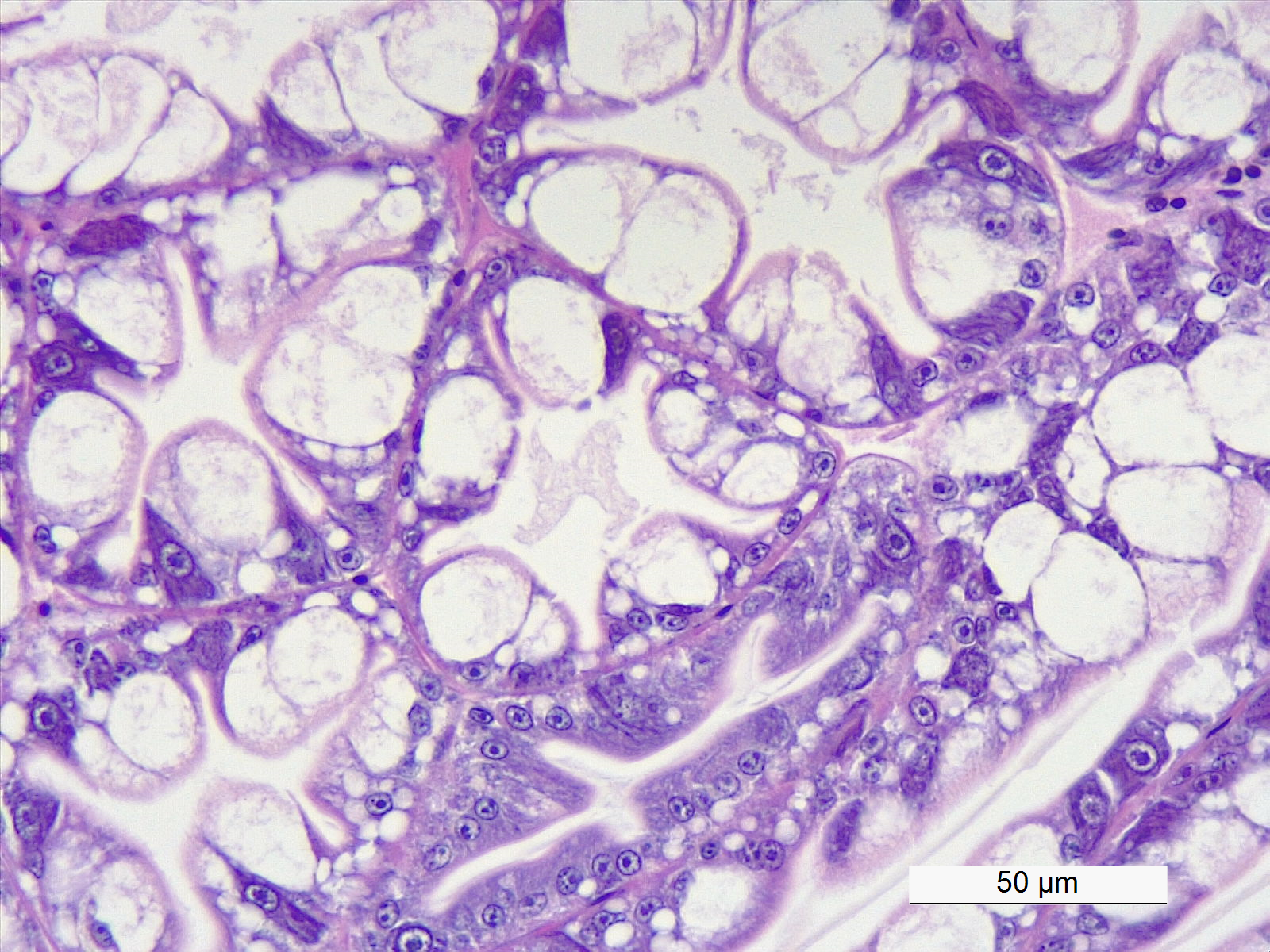

Supplement: S4 Raw images — (ZIP) [file pone.0261289.s004.zip › Fig 4B raw.tif]

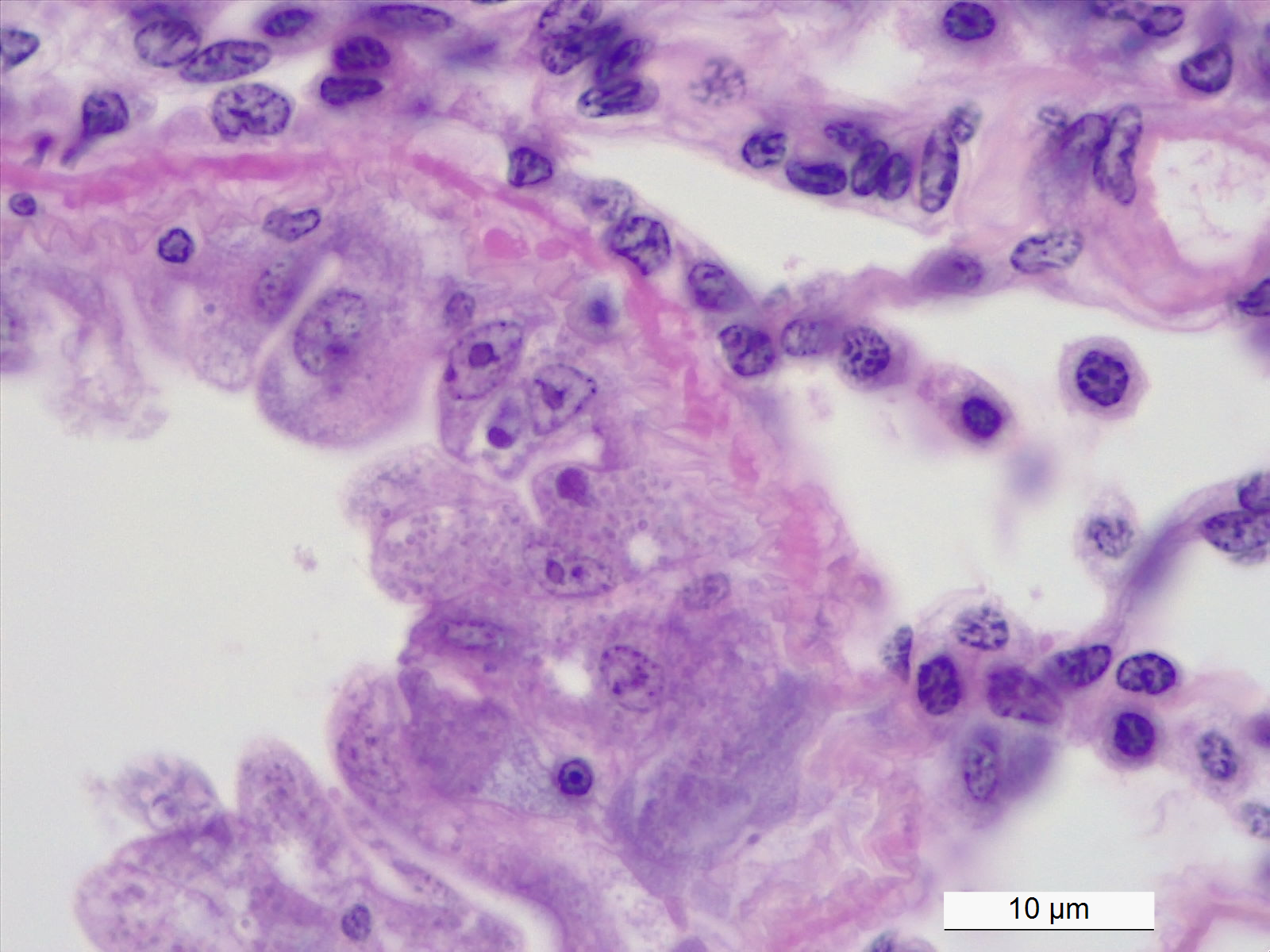

Supplement: S4 Raw images — (ZIP) [file pone.0261289.s004.zip › Fig 4C raw.tif]

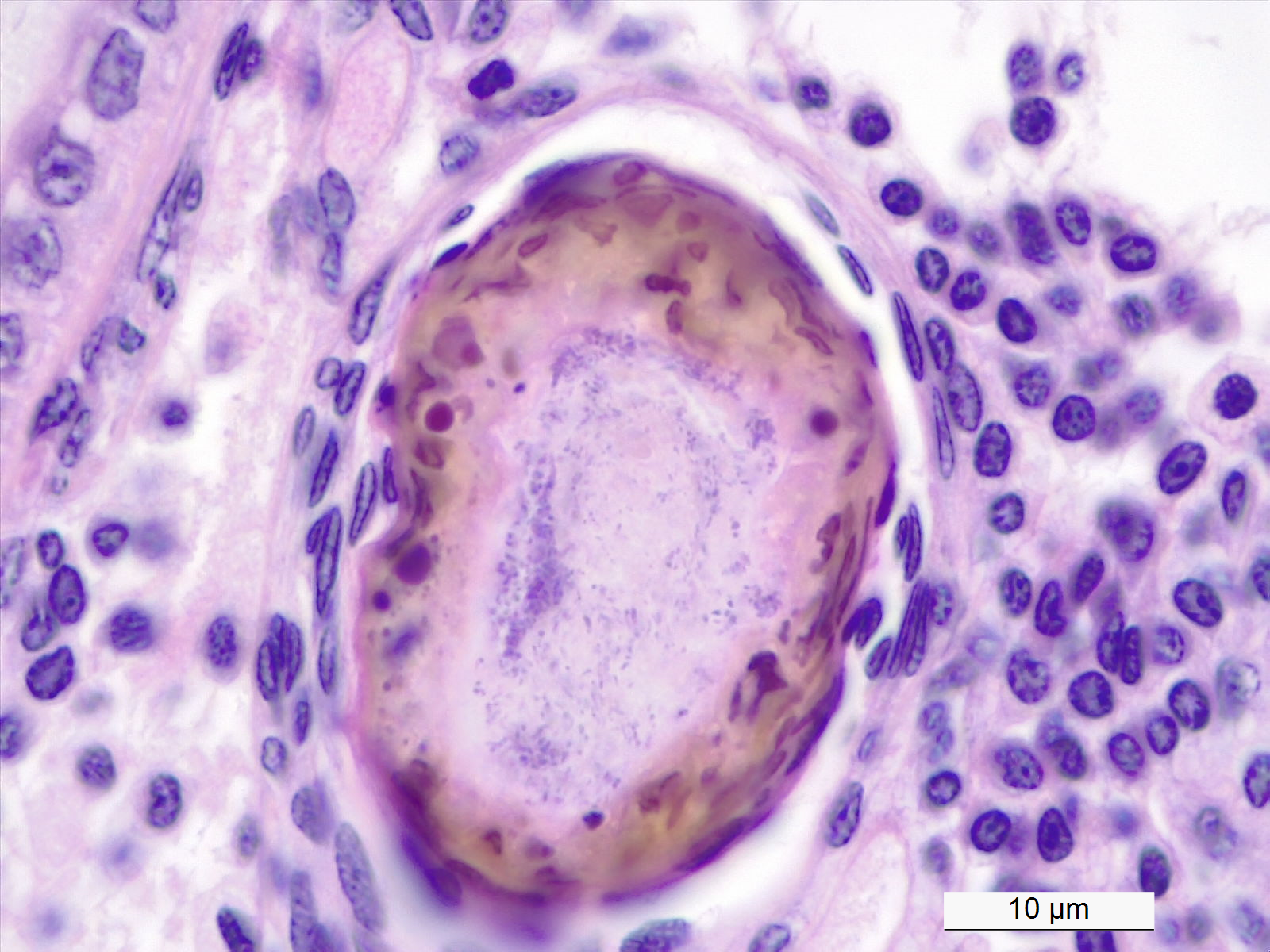

Supplement: S4 Raw images — (ZIP) [file pone.0261289.s004.zip › Fig 4D raw.tif]

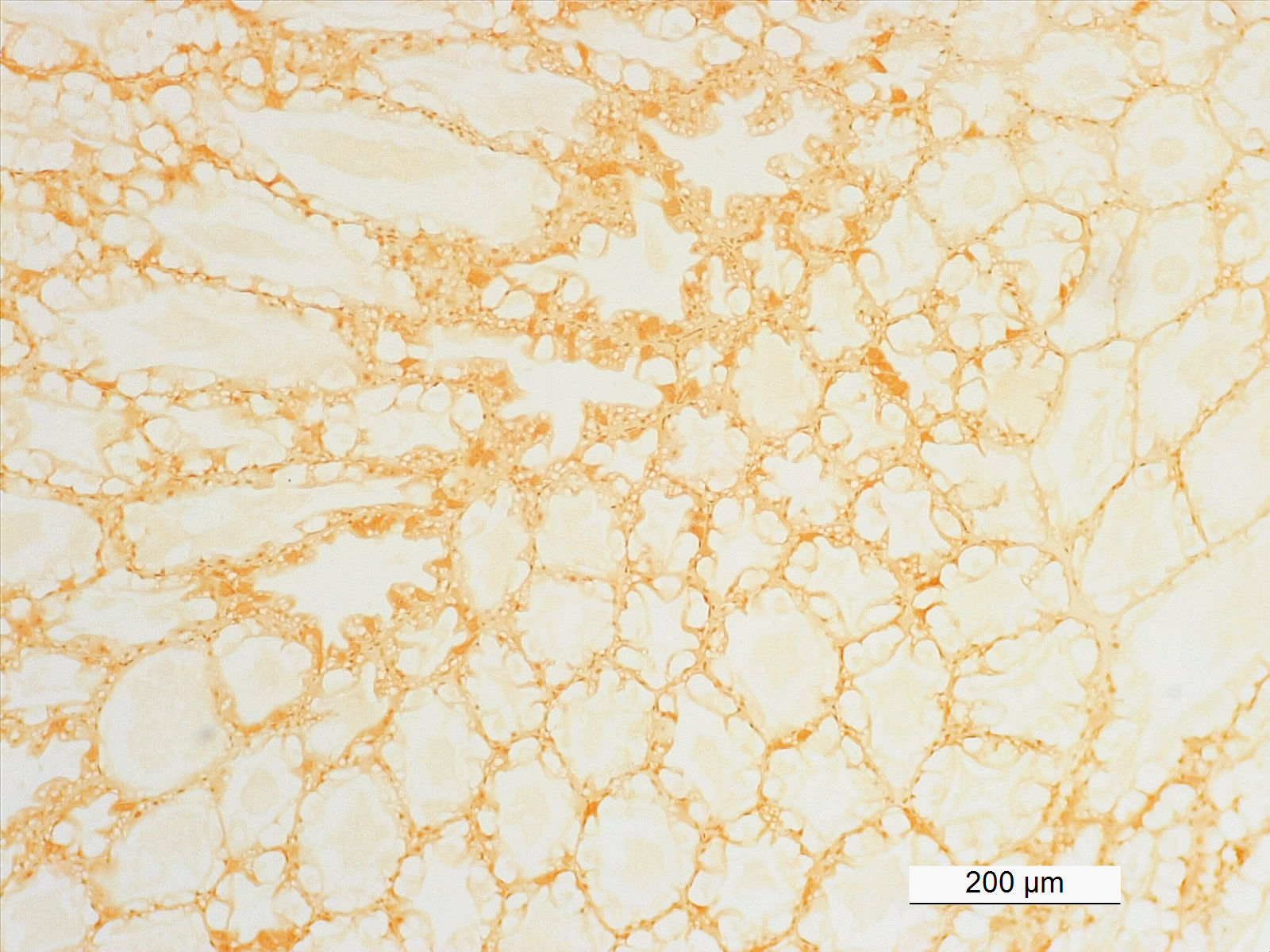

Supplement: S5 Raw images — (ZIP) [file pone.0261289.s005.zip › Fig 5A raw.tif]

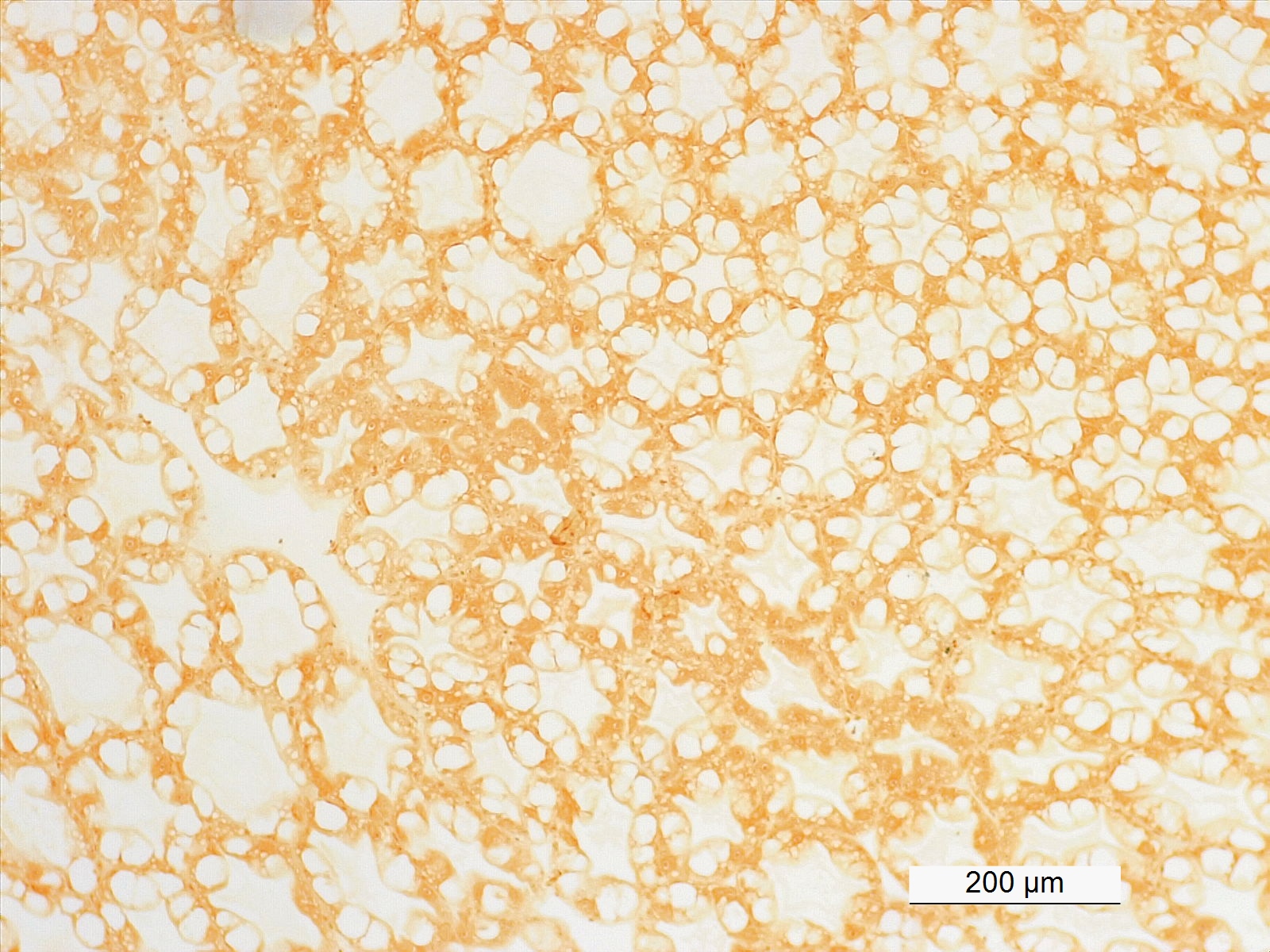

Supplement: S5 Raw images — (ZIP) [file pone.0261289.s005.zip › Fig 5B raw.tif]

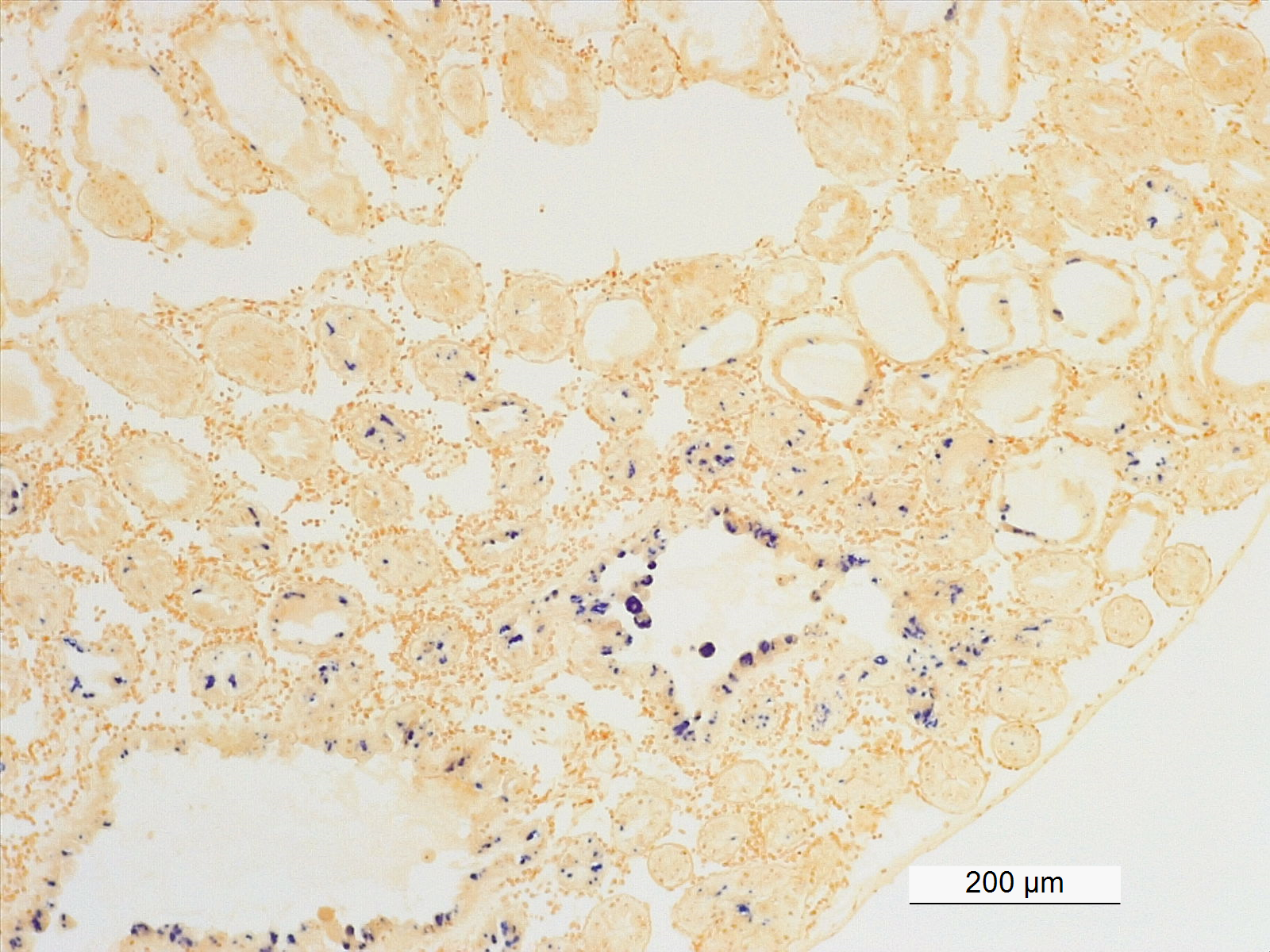

Supplement: S5 Raw images — (ZIP) [file pone.0261289.s005.zip › Fig 5C raw.tif]

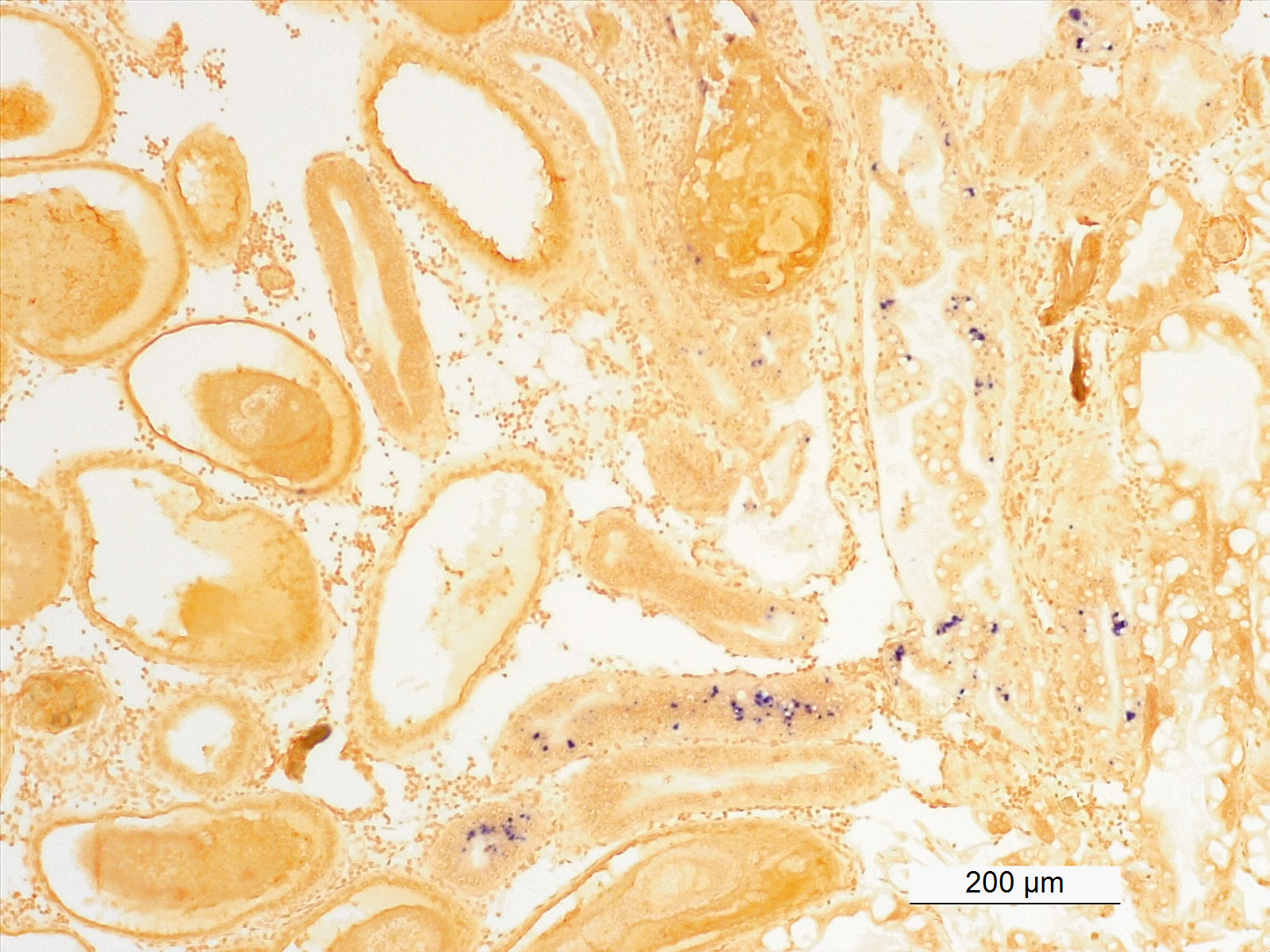

Supplement: S5 Raw images — (ZIP) [file pone.0261289.s005.zip › Fig 5D raw.tif]
